# Non-infectious pediatric uveitis in a single french tertiary center

**DOI:** 10.1186/1546-0096-12-S1-P233

**Published:** 2014-09-17

**Authors:** Dalila Nouar, Cyrille Hoarau, Florence Uettwiller, Marie-Laure Le Lez

**Affiliations:** 1 Immunology-allergology Unit, Bretonneau University Hospital, Tours, France; 2 Pediatric Rheumatology Unit, Clocheville University Hospital, Tours, France; 3 Ophtalmology Unit, Bretonneau University Hospital, Tours, France

## Introduction

Pediatric uveitis is a blinding disease in 15-20 % of cases. Juvenile idiopathic arthritis (JIA) is the most common disease associated with uveitis (JIAU).Biologics have changed the treatment of uveitis in particular JIAU.

## Objectives

To describe epidemiological, clinical, ophthalmologic and therapeutic characteristic of non infectious pediatric uveitis followed in our center.

## Methods

We retrospectively collected data between 2004 and 2013 for children under 16 years of age followed for non-infectious uveitis in the Department of Ophthalmology and in Pediatric Rheumatology Unit of the Hospital of Tours.

## Results

Of 32 children included there were 62 % of females and the mean age at diagnosis was 7.7 ± 3.4 years and 6.5 ± 3.8 years for JIAU. Ophthalmologic complications were present at the diagnosis in 72% of patients. Uveitis was unilateral in 56 % of cases and anterior in 75 % of cases.At the time of the last follow-up 41% were idiopathic uveitis and 41% JIAU. The number of inflammatory cells in the uvea (Tyndall) was improved in 50 % of cases and stabilized in 50 % of cases. One third of patients had presented new ophthalmologic complications. Uveitis was recurrent in 84 % of cases and 75% of uveitis had relapsed at least once. Local corticosteroid was always used in combination with systemic treatment . Corticotherapy was used in 41% of cases, and associated to DMARDS (methotrexate) in 19% of patients. Anti-TNFα (adalimumab) was used in 42% of patients and 54% of JIAU. An improvement in the visual acuity of our cohort was found (4/10 initially versus 6.3/10). In the subgroup of JIAU (13 patients) visual acuity was modified from 4/10 to 5/10 and 23% patients used the systemic association;corticosteroid and methotrexate , 3 patients relapsed under adalimumab.

## Conclusion

The risk of visual impairment in child uveitis is important. JIA is the main etiology.Biologics prescription have not been validated yet. The lack of consensus on the therapeutic strategy validate the need for a multidisciplinary collaboration between the ophthalmologist and the pediatric rheumatologist for a better management of children.

## Disclosure of interest

None declared.

